# Clostridium Bifermentans Infection of a Prosthetic Knee Joint in a Patient With Human Immunodeficiency Virus: A Case Report

**DOI:** 10.7759/cureus.47370

**Published:** 2023-10-20

**Authors:** Matthew Moran, Saloni H Patel, Gunjan Kahlon

**Affiliations:** 1 Internal Medicine, Einstein Medical Center Philadelphia, Philadelphia, USA; 2 Internal Medicine, Global Remote Research Scholars Program, Philadelphia, Ahmedabad, IND

**Keywords:** septic arthritis, human immunodeficiency virus, hiv, prosthetic knee joint infection, paraclostridium bifermentans, clostridium bifermentans

## Abstract

We reported a case of *Clostridium bifermentans* (*C. bifermentans*) infection in the prosthetic knee joint of a human immunodeficiency virus (HIV) patient, who presented with swelling, discomfort, pain, and redness in the right lower extremity. An uncommon yet potentially lethal human illness triggered by *C. bifermentans*. Foreign material is especially susceptible to local infection because of the local immunodeficiency close to the implant. Intravenous (IV) cefepime and IV ampicillin/sulbactam were administered to the patient. The idea of performing surgery to eradicate the infection was under consideration, but its necessity remained uncertain, and the decision to proceed with surgery had not been finalized.

## Introduction

The Gram-positive bacillus *Clostridium bifermentans*, reclassified as *Paraclostridium bifermentans*, is rod-shaped, anaerobic, gas-producing, sulfite-reducing, motile, endospore-forming, and capsulated [1]. In addition to the female vaginal canal and the oral cavity, cured meat, dry smoked sausages, cooked meat products, sewage, polluted water, feces, and marine sediments all contain it. It has the ability to withstand extreme temperatures, radiation, desiccation, poisonous chemicals, and other environmental stressors by existing as dormant spores. Of this group, *C. bifermentans* is often a non-pathogenic component that makes up a minor portion of the gut flora. From the literature review, this bacterium can lead to necrotizing endometritis, empyema, metastatic osteomyelitis, endocarditis, soft tissue infection, brain abscess, lymphadenitis, bacteremia, and panophthalmitis [2-7]. A study of the literature revealed one incidence of septic infection of a prosthetic joint in a 67-year-old man that was brought on by an isolated *C. bifermentans* and a case of septic arthritis of the knee joint due to *C. bifermentans* [8-9]. We present the first instance of prosthetic joint infection (PJI) in a human immunodeficiency virus (HIV) patient caused by isolated *C. bifermentans*.

## Case presentation

A 61-year-old African-American man with a medical history of HIV for 20 years, generalized erythroderma, right total hip arthroplasty in 2001 (22 years ago), right total knee arthroplasty in 2009 (14 years ago), and chronic distal femoral fracture status post open reduction and internal fixation (ORIF) without a history of hardware complication presented to the emergency department with complaints of right lower extremity swelling from foot to mid-thigh associated with redness and pain, along with subjective fever and chills since 7 days.

**Figure 1:**
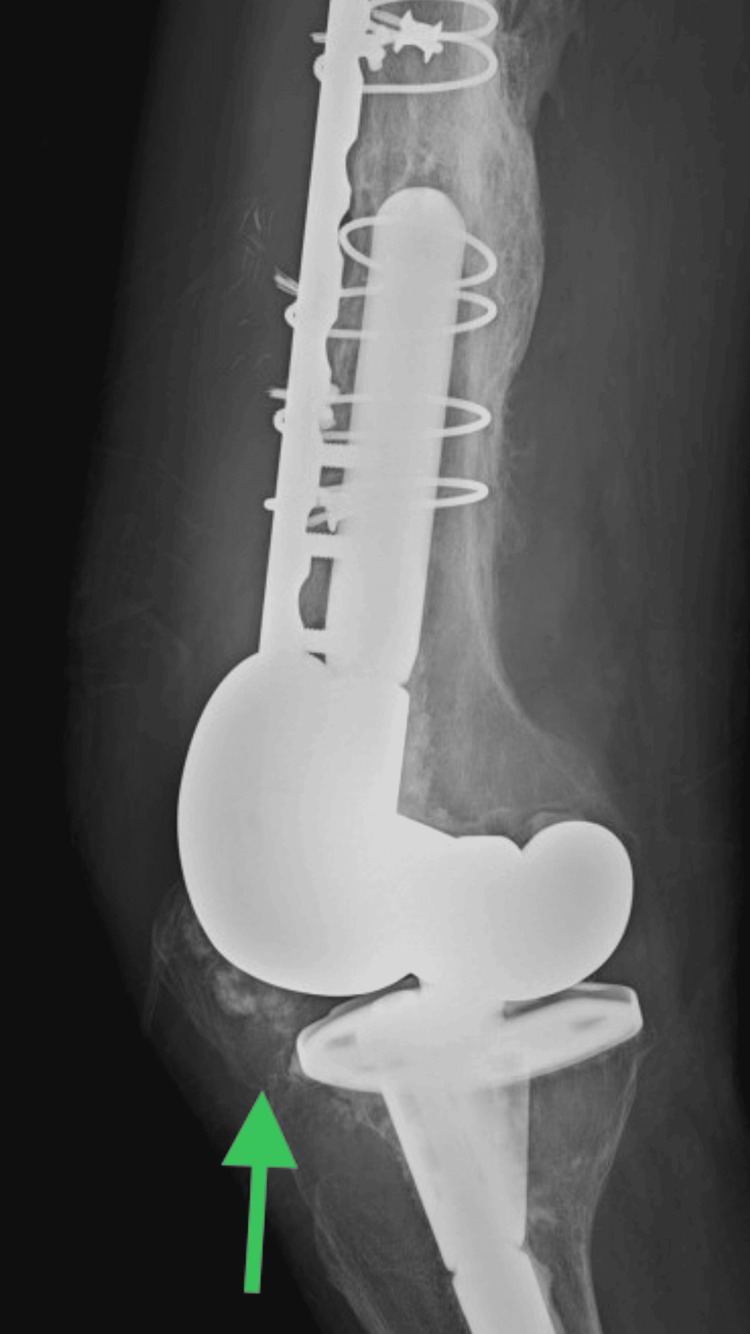
Right knee X-ray The right knee X-ray revealed no joint effusion and suboptimal prepatellar soft tissue edema (Green arrow). X-ray is not very significant.

The patient was hemodynamically stable when he first presented to the emergency room. Leukocytosis of 12.7 x 109/L, which subsequently climbed to 25.2 x 109/L within 12 days, hemoglobin of 9.2 g/dL, platelet count of 466 x 103/mcL, and raised erythrocyte sedimentation rate (ESR) and C-reactive protein (CRP) were all found during the laboratory evaluation. The right knee X-ray revealed no joint effusion and suboptimal prepatellar soft tissue edema, a complete knee replacement, chronic distal femoral fracture status following ORIF, and no signs of hardware complications (Figure 1). The patient was admitted for broad-spectrum intravenous (IV) antibiotic treatment with vancomycin and cefazolin, as well as IV fluids, due to his history of immunosuppression. Bactrim orally (PO) was also administered. Following consultation with the infectious diseases experts, vancomycin was maintained as advised. Blood culture was negative.

**Figure 2:**
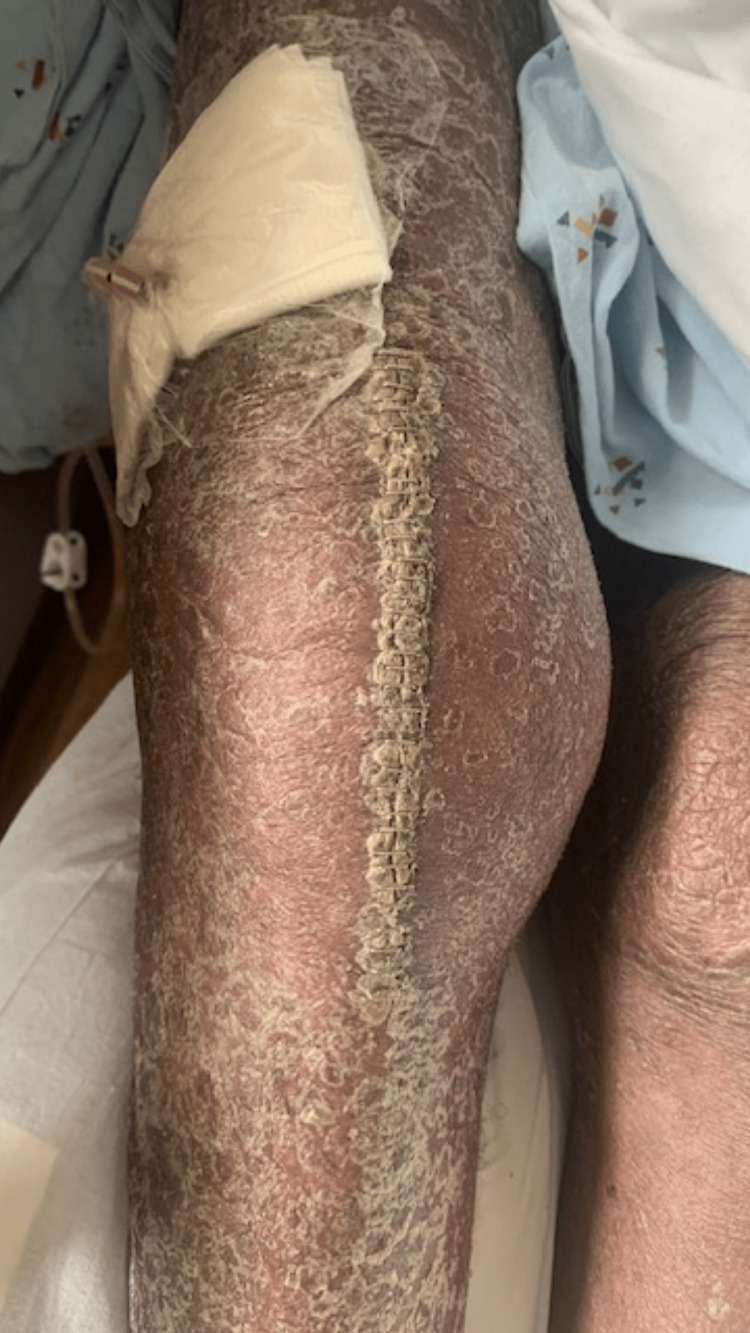
Right knee swelling with a drain in place

Right knee arthrocentesis was performed on the third day of hospitalization, and fluid was collected and sent for a culture of bacteria, fungi, and acid-fast bacilli. Upon fluid analysis, 3074 cells/mcL white blood cells and 79% segmented cells were found (Table 1). The lower extremity angiography that was performed on the sixth day was normal. To enable continuous draining of the fluid, an interventional radiologist (IR) implanted a drain (Figure 2) on the 9th day. The fluid aspirated from the right knee joint tested positive for *C. bifermentans*, but after 10 days, the test became negative. According to the infectious diseases team’s advice, Bactrim was withdrawn and IV cefepime and IV ampicillin/sulbactam were added. The orthopedic surgery team performed irrigation and debridement on the right knee, performing sharp excisional debridement down to the level of the bone and joint and inserting a Hemovac drain into the right knee joint.

**Table 1:** Laboratory findings and knee joint fluid analysis mcL: microliter; g/dL: gram/deciliter

Laboratory findings	Result	Reference value
Blood white blood cell count	12.7 x 10^3^/mcL	4.5-11.0 × 10^3^/mcL
Blood hemoglobin	9.2 g/dL	13.8-17.2 g/dL
Blood platelet count	466 x 10^3^/mcL	150-450 x 10^3^/mcL
Joint fluid white blood cell count	3074 cells/mcL	<200 cells/mcL
Joint fluid segmented cell percentage (%)	79%	<25%
Erythrocyte sedimentation rate (ESR)	124 mm/h	0-15 mm/h
C-reactive protein (CRP)	183 mg/L	<3 mg/L

**Figure 3:**
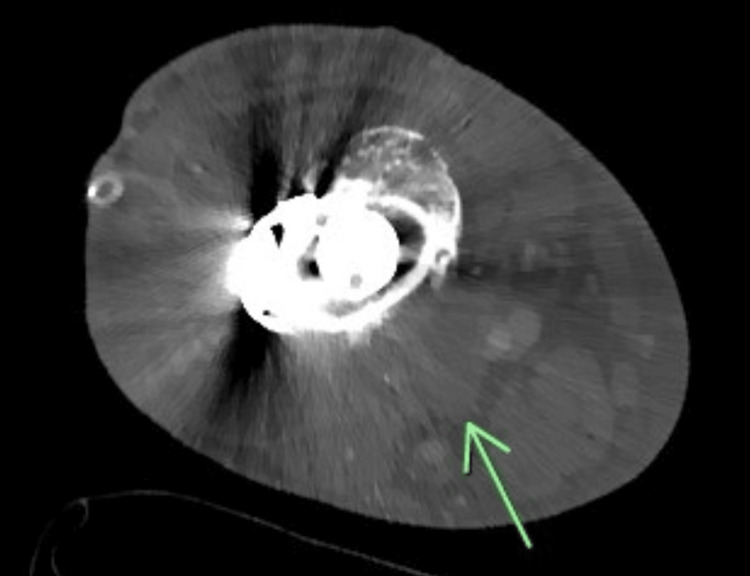
CT scan of the right lower extremity A right lower extremity CT scan with contrast revealed fluid collection with rim enhancement that was consistent with an abscess around the posterior and lateral portions of the right distal femur (Green arrow).

On the 12th day, a right lower extremity CT scan with contrast revealed fluid collection with rim enhancement that was consistent with an abscess around the posterior and lateral portions of the right distal femur (Figure 3). A 3cc sample of the fluid from the prophylactic right hip arthrocentesis was sent for testing and culturing. Fluids from the right hip had negative cultures. After a few days, the drain was changed and the option of surgery to remove the prosthesis was considered, but it was not a definite decision.

## Discussion

Medical device-related infections, such as PJI, can develop due to biofilm formation. The creation of biofilms explains how typical flora organisms can turn pathogenic when they grow near foreign objects, necessitating surgical intervention. The implicated organisms in delayed- and late-onset PJIs are concentrated on the prosthesis’ surface, which limits the sensitivity of periprosthetic tissue and fluid cultures. To get around this restriction, you may use a technology like vortexing-sonication to sample the prosthetic surface directly [10]. The Infectious Diseases Society of America suggests a 4-6-week course of pathogen-specific IV or highly bioavailable oral antibiotic treatment [11]. Due to usually reduced soft tissue coverage and an undamaged joint capsule that may limit continuous infection spread, it is practically more appealing to do irrigation and debridement on an infected total knee arthroplasty.

In our HIV patient with *C. bifermentans* infection of a prosthetic knee joint, IV cefepime, and IV ampicillin/sulbactam were administered to the patient. Surgery for prosthesis explantation was considered, but it was not a definite decision due to the absence of data. In one of the case reports, an 18-year-old’s septic arthritis of the knee developed 13 days after the meniscectomy procedure was performed on the knee. In this instance, the patient had an arthrotomy, had the joint debrided, and received intra-articular and IV penicillin therapy for a week. After that, the patient received a two-week prescription for oral penicillin [9]. Since PJI with *C. bifermentans* has never been documented before, there is no established course of treatment. A 67-year-old man who had *C. bifermentans*-caused septic arthritis of the knee joint underwent a thorough washout and debridement (DAIR) of the joint while keeping his prosthesis. He received oral suppression with amoxicillin-clavulanic acid for 6 months after receiving IV ampicillin-sulbactam for 2 weeks [8].

Multiple irrigation and debridement treatments, as well as prolonged antibiotic treatment, may increase success. Gram-positive infections, the absence of sinus formation, the use of antibiotic-impregnated bone cement for fixing the new prosthesis, and long-term antibiotic medication were all factors linked to successful direct exchange arthroplasty. A relative contraindication to direct exchange arthroplasty is an immunocompromised host [12]. Patients who experience DAIR failure ultimately have a two-stage arthroplasty exchange. Late chronic infections would benefit from a two-stage arthroplasty exchange. Between the first and second stages, the majority of patients are given IV antibiotics that are pathogen-directed for 4 to 6 weeks [10]. In the case of our patient, the possibility of surgery to remove the prosthesis was deliberated, but it remained uncertain.

## Conclusions

Knee joint infections, especially those involving prosthetic joints, should be differentially diagnosed for a *C. bifermentans* infection. IV broad-spectrum antibiotics should be administered to the patient at first, and based on the culture results, medications should be selected more specifically based on the organism. In our case involving an HIV patient with an infection focus, we were contemplating the possibility of performing knee joint prosthesis removal surgery as a means to eliminate the source of the infection, but surgery was not definite. To prevent a recurrence and ensure successful treatment, the patient may need to receive long-term IV antibiotic therapy in the event of surgery.
